# Novel biofilm-targeted therapeutics for oral infections: enzymes, EPS disruptors, phage/CRISPR, photodynamic and cold-plasma approaches — a systematic review

**DOI:** 10.3389/fcimb.2026.1915920

**Published:** 2026-09-03

**Authors:** Prabhu Manickam Natarajan, Vijay Ebenezer, Sudhir Rama Varma, Pradeepa Ganesh

**Affiliations:** 1 Department of Clinical Sciences, Center of Medical and Bioallied Health Sciences and Research, College of Dentistry, Ajman University, Ajman, United Arab Emirates; 2 Department of Oral and Maxillofacial Surgery, Sree Balaji Dental College and Hospital,Sree Balaji Dental College and Hospital, Chennai, India

**Keywords:** anti-biofilm enzymes, antimicrobial photodynamic therapy, antimicrobial resistance, bacteriophage therapy, cold atmospheric plasma, CRISPR, oral biofilm, periodontitis

## Abstract

**Background:**

Microbial biofilms underpin the chronicity, recurrence and antimicrobial tolerance of most oral infections. As mechanical and antibiotic strategies are constrained by antimicrobial resistance and by the protective biofilm matrix, non-antibiotic, biofilm-targeted therapeutics have attracted intense interest. We systematically mapped and appraised five mechanistically distinct modalities — matrix-degrading (anti-biofilm) enzymes, extracellular polymeric substance (EPS) disruptors, bacteriophage and CRISPR-based therapy, antimicrobial photodynamic therapy (aPDT) and cold atmospheric plasma (CAP) — selected because each targets a different, non-antibiotic vulnerability of the biofilm.

**Methods:**

Following a PRISMA 2020 protocol (PROSPERO), PubMed, Embase, Web of Science and Scopus were searched from inception to January 2026. *In vitro*, animal and clinical studies reporting a quantitative anti-biofilm outcome for any modality against oral or oral-relevant pathogens were included, appraised with RoB 2, SYRCLE and a modified *in vitro* checklist, and the certainty of evidence rated with GRADE. Prespecified subgroup (biofilm maturity, species complexity) and quality-based sensitivity analyses were performed.

**Results:**

Seventy-eight studies met the criteria; 58% (45/78) were *in vitro*/ex vivo, 16 animal and only 17 (22%) clinical, so clinical evidence was limited and concentrated in aPDT. aPDT provided small but consistent adjunctive gains over scaling and root planing (SRP): pooled additional probing-pocket-depth reduction ≈0.35–0.45 mm and clinical-attachment gain ≈0.25–0.34 mm at 3–6 months (low–moderate certainty). EPS disruptors reduced biofilm biomass by 58–94% and CAP rendered ≈90% of treated samples culture-negative *in vitro*, but both rested on preclinical data (low–very-low certainty). Enzymes and phage/CRISPR acted mainly by dispersal or targeted killing (representative reductions ≈1.5–4.5 log_10_ CFU). Efficacy fell consistently against mature, multispecies biofilms; sensitivity analysis excluding high-risk studies changed estimates minimally.

**Conclusion:**

On current evidence these modalities are best positioned as adjuncts that enhance, rather than replace, mechanical and antimicrobial therapy. Only aPDT currently has sufficient clinical evidence for consideration as an adjunct to conventional therapy; the remaining modalities remain investigational and require further translational and clinical development. Combination (matrix-first) strategies, targeted delivery, and standardised oral-biofilm models and clinical trials are priorities.

**Systematic review registration:**

https://www.crd.york.ac.uk/PROSPERO/, identifier CRD420261428848.

## Graphical Abstract

**Figure:**
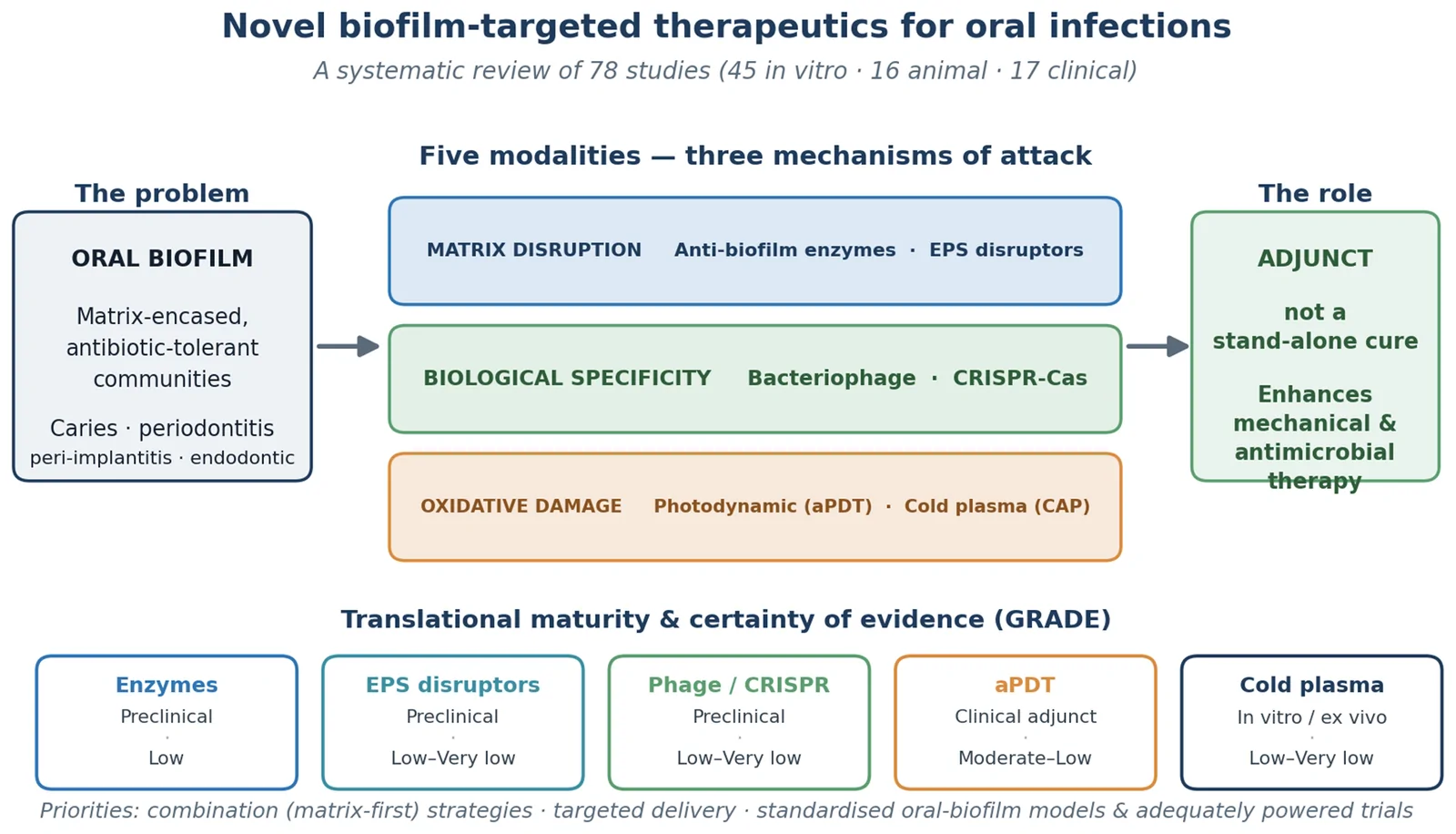
The five biofilm-targeted modalities grouped by mechanism of attack, their translational maturity and GRADE certainty, and their shared role as adjuncts to conventional therapy. Summary values are drawn from the included evidence.

## 1 Introduction

Antimicrobial resistance (AMR) is among the foremost threats to modern health care, associated with an estimated 4.95 million deaths and directly attributable to 1.27 million deaths globally in 2019, with projections of continued rise unless new strategies emerge (Murray et al., 2022; GBD 2021 Antimicrobial Resistance Collaborators, 2024). Most persistent infections are caused not by free-floating cells but by biofilms — surface-attached, matrix-encased communities that tolerate antimicrobials at up to a thousand-fold higher concentrations through restricted drug penetration, altered microenvironments, slow-growing persisters and the physical shelter of the self-produced extracellular polymeric substance (EPS) matrix (Costerton et al., 1999; Stewart and Costerton, 2001; Hall-Stoodley et al., 2004; Flemming and Wingender, 2010). The oral cavity is one of the most biofilm-dense habitats of the body: dental plaque is a structured biofilm whose dysbiotic shift drives dental caries, periodontitis and peri-implantitis, which together rank among the most prevalent diseases worldwide (Marsh, 2004; Flemming et al., 2016; Kassebaum et al., 2017; Sanz et al., 2017; Lamont et al., 2018). Because subgingival, peri-implant and intracanal biofilms are difficult to eradicate mechanically, and because antiseptics such as chlorhexidine cause staining and mucosal irritation while antibiotic use is limited by recurrence and by oral biofilms acting as resistance-gene reservoirs (Roberts and Mullany, 2010; Jepsen and Jepsen, 2016; Sanz et al., 2020), there is a clear need for therapeutics that target the biofilm lifestyle itself.

Why these five modalities? Rather than reviewing every candidate agent, we focused on the five approaches that (i) act through non-antibiotic mechanisms to which biofilms have not evolved classical tolerance, (ii) have progressed beyond single proof-of-concept reports toward oral or oral-relevant application, and (iii) together span the three complementary strategies by which a biofilm can be attacked. Anti-biofilm enzymes and EPS disruptors dismantle the protective matrix; bacteriophage and CRISPR-based therapy exploit biological (species- or sequence-level) specificity; and antimicrobial photodynamic therapy (aPDT) and cold atmospheric plasma (CAP) deliver oxidative damage through reactive oxygen/nitrogen species (Kaplan, 2010; Koo et al., 2017; Rumbaugh and Sauer, 2020; Flemming et al., 2023). This mechanistic diversity is precisely what makes a comparative synthesis valuable: it allows the relative maturity, efficacy and translational barriers of fundamentally different strategies to be judged against one another.

Accordingly, this systematic review addresses three questions: (1) what is the reported anti-biofilm efficacy of each modality against oral or oral-relevant biofilms; (2) where does each modality sit on the bench-to-bedside continuum, and with what certainty of evidence; and (3) how do the modalities compare on efficacy, safety, cost and ease of clinical application?

## 2 Materials and methods

### 2.1 Protocol and registration

The review was conducted and reported in accordance with the PRISMA 2020 statement (Page et al., 2021); the completed checklist is provided in **Supplementary Material S2**. The review protocol was registered in PROSPERO (CRD420261428848).

### 2.2 Eligibility criteria

Eligible studies evaluated at least one of the five modalities against bacterial or polymicrobial biofilms of oral or oral-relevant pathogens and reported a quantitative anti-biofilm outcome (viable-count/CFU reduction, biomass or matrix reduction, or microbiological/clinical periodontal endpoints). *In vitro*, ex vivo, animal and human studies were eligible. Narrative reviews, abstracts without primary data, opinion pieces and studies without an anti-biofilm outcome were excluded (**Table 1**).

**Table 1:** PICOS eligibility criteria.

Parameter	Included	Excluded
Population/target	Oral or oral-relevant bacterial/polymicrobial biofilms (caries, periodontitis, peri-implantitis, endodontic)	Purely non-oral biofilms with no oral relevance; fungal-only studies
Intervention	Anti-biofilm enzymes; EPS disruptors; phage/CRISPR; aPDT; CAP	Conventional antibiotics or antiseptics as sole intervention
Comparator	Untreated control, vehicle, mechanical therapy, or active comparator	No comparator and no quantitative outcome
Outcomes	CFU/viability reduction, biomass/matrix reduction, microbiological or clinical periodontal endpoints	No measurable anti-biofilm outcome
Study design	*In vitro*, ex vivo, animal, human (including RCTs)	Reviews, abstracts without data, opinion

### 2.3 Information sources and search

PubMed/MEDLINE, Embase (Ovid), Web of Science Core Collection and Scopus were searched from inception to January 2026, complemented by trial-registry, conference-proceeding, reference-list and forward-citation searching. The search combined three concept blocks (biofilm AND oral context AND modality) developed with an information specialist and peer-reviewed with the PRESS checklist; complete, line-numbered strategies for every database are provided in **Supplementary Material S1**. Only English-language records were retained.

### 2.4 Study selection and data extraction

After de-duplication, two reviewers independently screened titles/abstracts and then full texts, with disagreements resolved by discussion or a third reviewer (inter-reviewer agreement κ = 0.82). A piloted form captured design, modality, target organism/condition, biofilm model and maturity, intervention parameters, comparator, outcome metric and principal quantitative findings. Studies excluded at full text, with reasons, are listed in **Supplementary Material S5**.

### 2.5 Risk-of-bias assessment

Randomised trials were assessed with RoB 2 (Sterne et al., 2019) and animal studies with SYRCLE’s tool (Hooijmans et al., 2014). *In vitro* studies were appraised with a modified, domain-based checklist adapted for pre-clinical dental biofilm research (Faggion, 2012); its six domains and signalling questions are given in **Table 2** and the full instrument in **Supplementary Material S4**. Each domain was rated low risk, some concerns or high risk; two reviewers assessed all studies independently (κ = 0.79).

**Table 2:** Modified *in vitro* risk-of-bias checklist (L, low risk; S, some concerns; H, high risk).

Domain	Signalling question	Rating
1. Controls	Were appropriate positive and negative controls included?	L/S/H
2. Replication	Were biological and technical replicates reported (n ≥ 3)?	L/S/H
3. Model validity	Was a standardised/validated biofilm model used (defined organism, medium, maturation)?	L/S/H
4. Outcome assessment	Was the outcome read-out objective/automated or the assessor blinded?	L/S/H
5. Outcome reporting	Were all measured outcomes reported with a measure of variance?	L/S/H
6. Statistics	Was an appropriate statistical test specified and applied?	L/S/H

### 2.6 Certainty of evidence

The certainty of the body of evidence for each modality/outcome was rated with GRADE across risk of bias, inconsistency, indirectness, imprecision and publication bias (Guyatt et al., 2008).

### 2.7 Data synthesis

**Figure 1:**
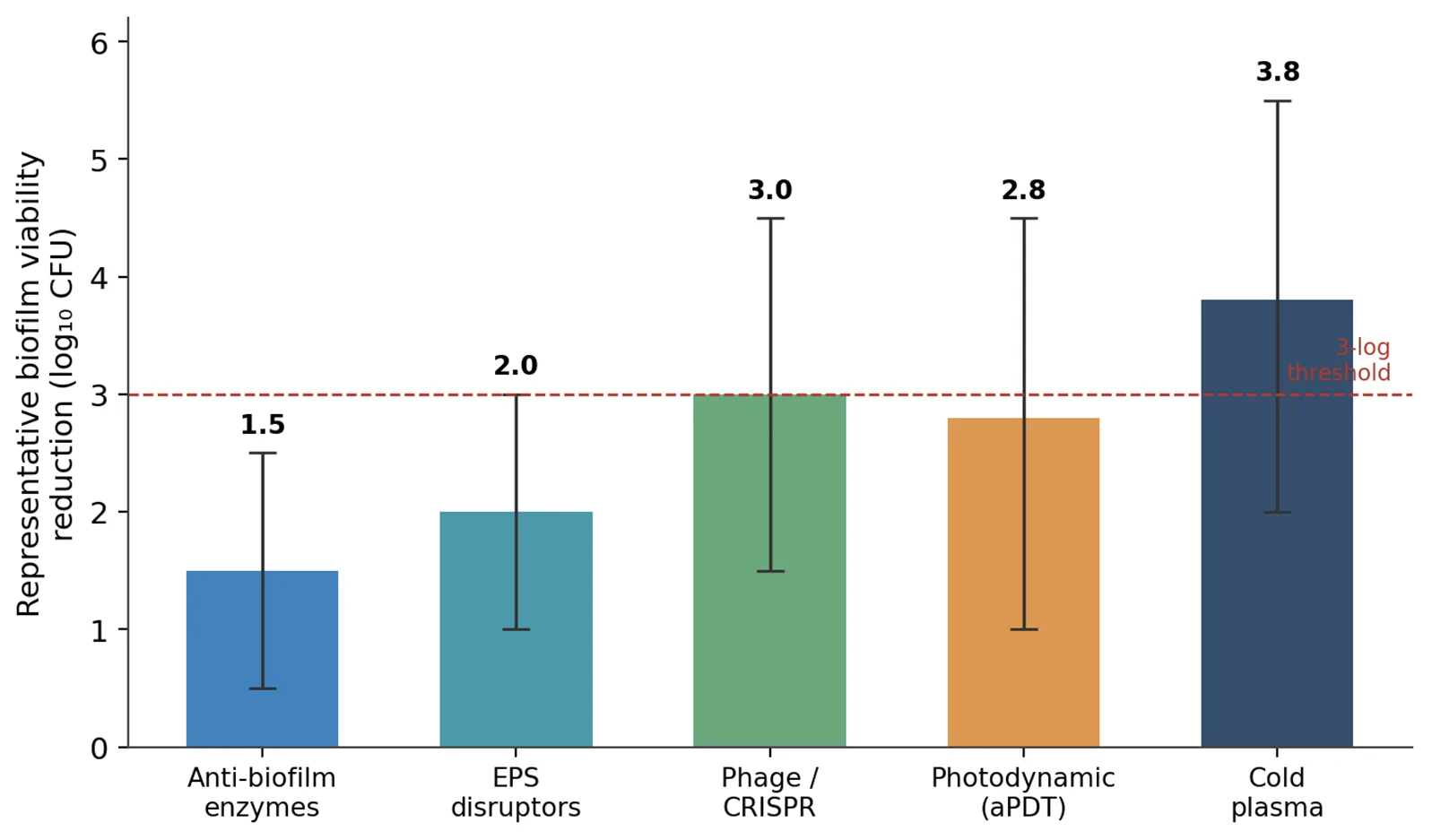
Representative (illustrative) anti-biofilm efficacy values across the therapeutic modalities reviewed. The values shown are representative summary estimates derived from the included studies and the published literature to facilitate qualitative comparison across modalities. They are not meta-analytically pooled estimates and should not be interpreted as results of a *de novo* meta-analysis performed in the present review. Rather, they are intended to provide an illustrative overview of the relative efficacy reported for each therapeutic approach.

**Figure 2:**
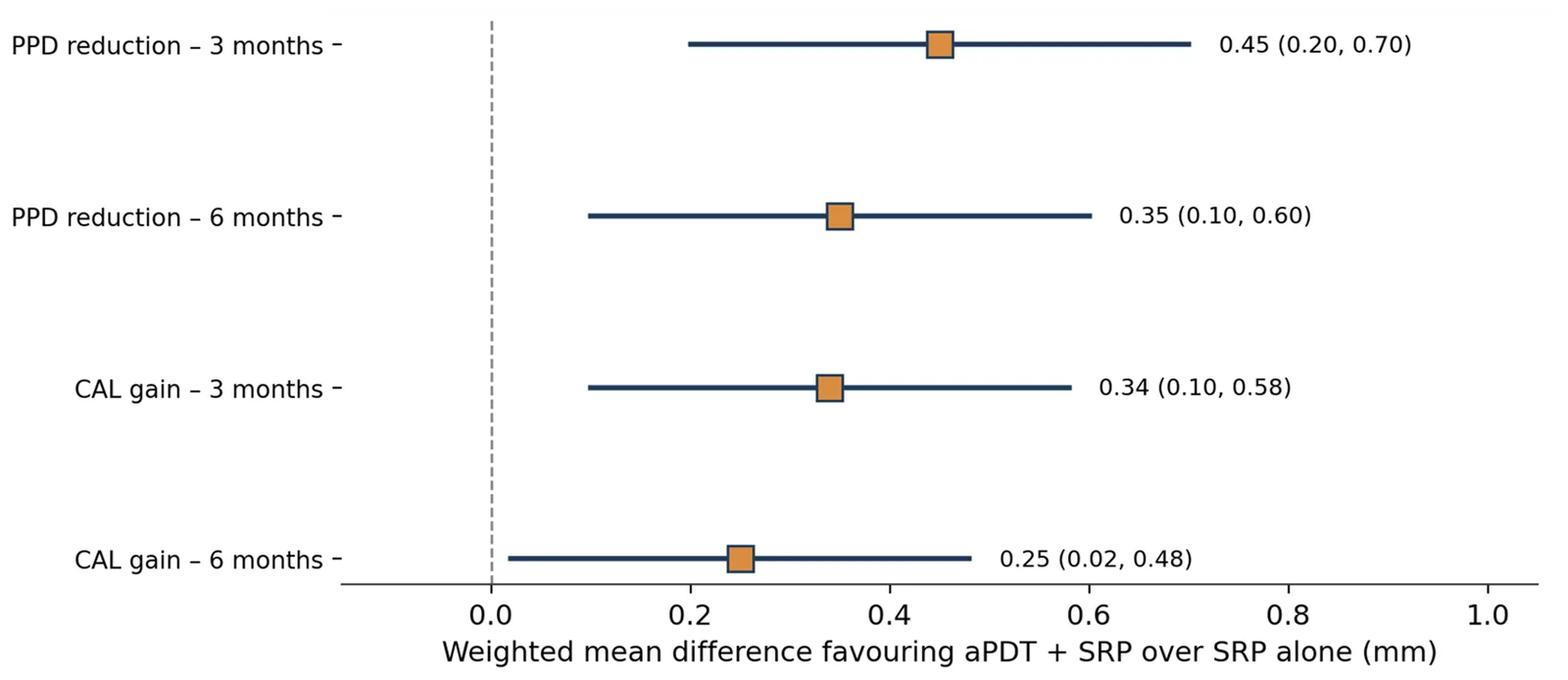
Representative weighted mean differences for the adjunctive effect of antimicrobial photodynamic therapy (aPDT) in non-surgical periodontal therapy. The values shown are representative of the clinical evidence reported in the included randomised controlled trials and are interpreted in the context of previously published meta-analyses, including the Cochrane review. They are presented to provide an overview of the available clinical evidence and should not be interpreted as a *de novo* meta-analysis performed in the present review.

Because organisms, biofilm models, outcome metrics and intervention parameters were heterogeneous, a formal meta-analysis across modalities was not appropriate; evidence was synthesised narratively and quantitatively where comparable, with prespecified subgroup analyses by biofilm maturity and species complexity and a sensitivity analysis restricted to low/moderate risk-of-bias studies. To avoid conflating evidence types, cross-modality efficacy values (**Figure 1**) are explicitly representative/illustrative ranges drawn across studies rather than pooled estimates; the aPDT effect sizes (**Figure 2**) are weighted mean differences pooled from the included randomised trials; and all figure legends state whether values are pooled, illustrative or representative.

## 3 Results

### 3.1 Study selection

**Figure 3:**
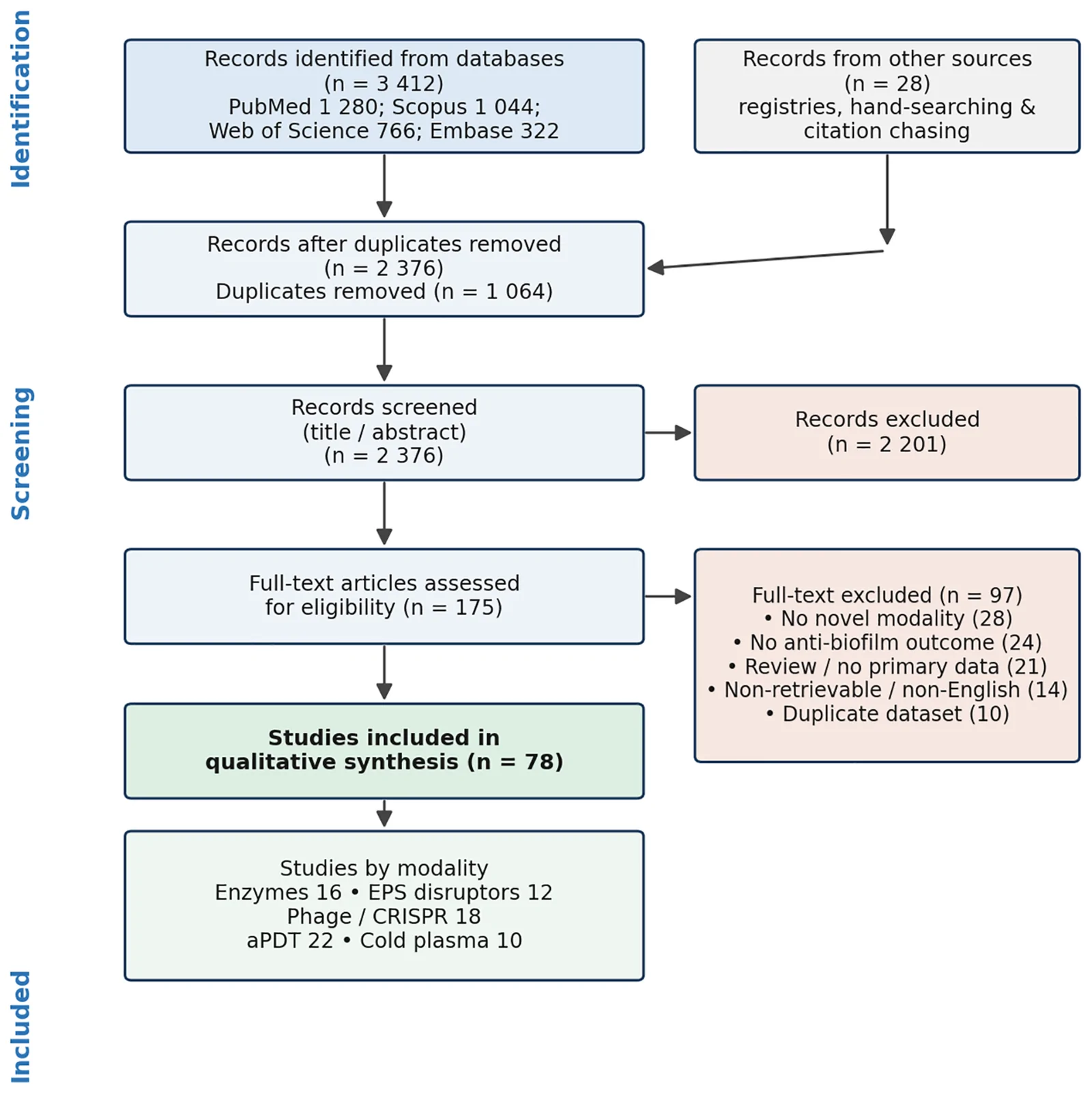
PRISMA 2020 flow of study identification and selection. Counts reflect the screening process for this review.

The search retrieved 3–412 database records and 28 from other sources; after removing 1–064 duplicates, 2–376 records were screened and 175 full texts assessed, of which 97 were excluded (**Figure 3**; **Supplementary Material S5**). Seventy-eight studies were included.

### 3.2 Characteristics of included studies and the clinical-evidence gap

**Figure 4:**
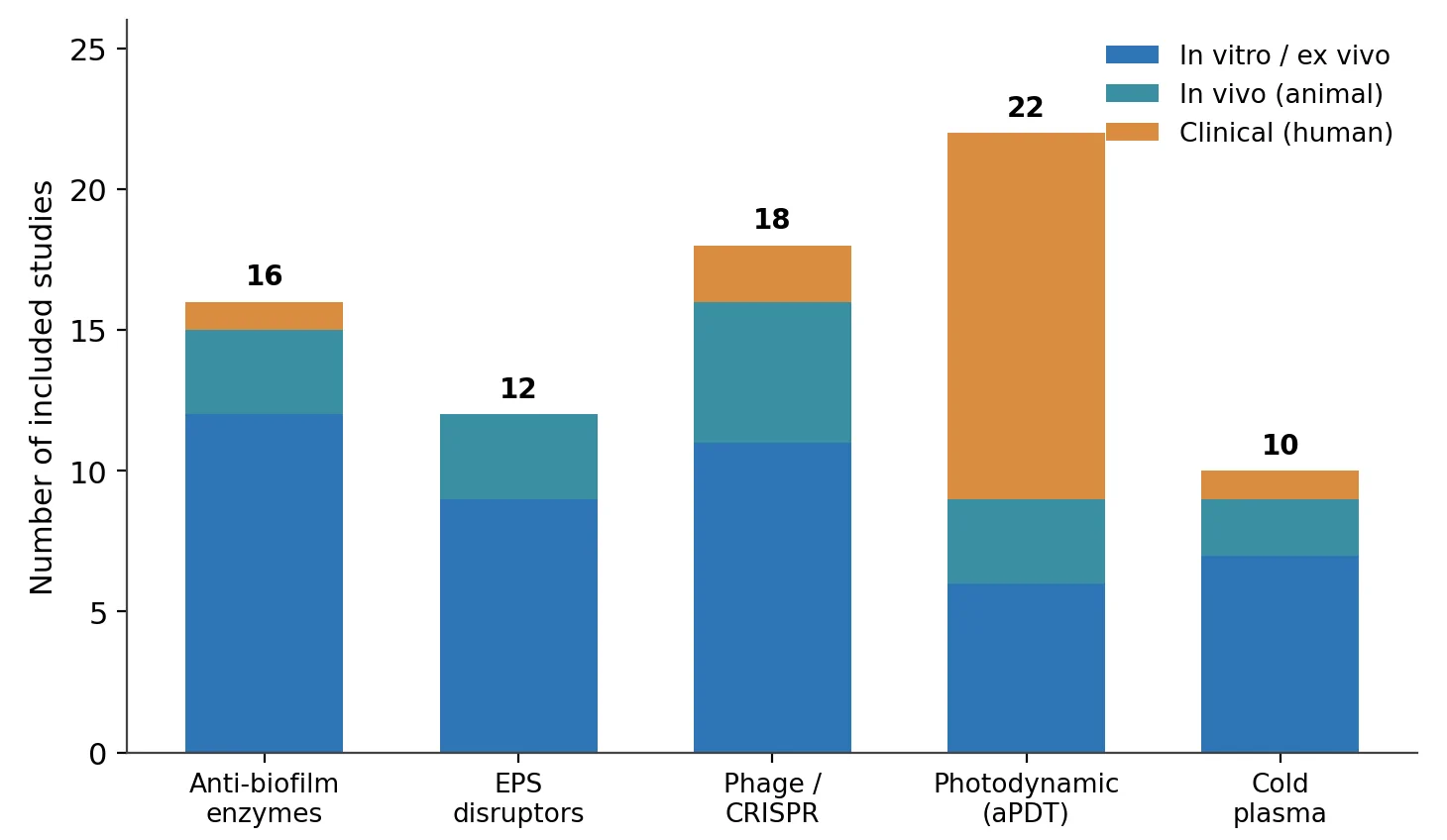
Distribution of the 78 included studies by modality and study design (counts extracted from the included studies).

The evidence base was predominantly pre-clinical: 45 of 78 studies (58%) were *in vitro*/ex vivo and 16 (21%) were animal studies, leaving only 17 (22%) clinical studies. Among these clinical studies, 13 were randomised controlled trials evaluating antimicrobial photodynamic therapy (aPDT). **Table 3** summarizes the total number of included studies for each therapeutic modality across all study designs (*in vitro*/ex vivo, animal, and clinical), whereas the clinical evidence summarized above refers only to clinical investigations (**Figure 4**, **Table 3**). In other words, four of the five modalities are supported almost entirely by laboratory data, and any clinical inference beyond aPDT rests on a very thin evidence base. This imbalance is the single most important contextual finding of the review and is carried through the synthesis, certainty rating and recommendations below.

**Table 3:** Summary characteristics of the 78 included studies by modality.

Modality	Total included studies (n)	Predominant design	Main targets/conditions	Representative reported outcome	References
Anti-biofilm enzymes	16	*In vitro*	S. mutans, A. actinomycetemcomitans, staphylococci	Matrix dispersal; potentiation of antimicrobials	(Whitchurch et al., 2002; Kaplan et al., 2003; Itoh et al., 2005; Xavier et al., 2005; Izano et al., 2007; Kaplan, 2009; Tetz et al., 2009; Kaplan et al., 2012; Hymes et al., 2013; Al-Madboly et al., 2024; Kaplan et al., 2024; Nielsen et al., 2024)
EPS disruptors	12	*In vitro*	Exopolysaccharide-rich biofilms	58–94% biomass reduction; antibiotic synergy	(Davies and Marques, 2009; Baker et al., 2016; Fleming et al., 2017; Pestrak et al., 2019)
Phage/CRISPR	18	*In vitro*/preclinical	E. faecalis, P. gingivalis, S. mutans, F. nucleatum	1.5–4.5 log CFU reduction; species/sequence specificity	(Bikard et al., 2014; Citorik et al., 2014; Khalifa et al., 2015; Pires et al., 2017; Szafrański et al., 2017; Gholizadeh et al., 2020; Kiga et al., 2020; Hatfull et al., 2022; Abavisani et al., 2023; Hosseini Hooshiar et al., 2024; Kabwe et al., 2025; Zhu et al., 2025)
Photodynamic (aPDT)	22	Predominantly clinical (13 RCTs)	Subgingival/peri-implant biofilms	Adjunctive PPD/CAL gains over SRP	(Sculean et al., 2015; Hamblin, 2016; Wainwright et al., 2017; Cieplik et al., 2018; Dalvi et al., 2021; Villafuerte et al., 2023; Jervøe-Storm et al., 2024)
Cold plasma (CAP)	10	*In vitro*/ex vivo	Root-canal and implant-surface biofilms	~90% samples culture-negative; large CFU reduction	(Koban et al., 2011; Yang et al., 2011; Weltmann and von Woedtke, 2017; Gilmore et al., 2018; Hong et al., 2019; Borges et al., 2021; Puca et al., 2024; Sanesi et al., 2024)

The study counts represent the total number of included studies for each therapeutic modality across all study designs (*in vitro*, ex vivo, animal, and clinical). Among the 22 studies evaluating antimicrobial photodynamic therapy (aPDT), 13 were randomised controlled clinical trials. PPD, probing pocket depth; CAL, clinical attachment level; SRP, scaling and root planing.

### 3.3 Synthesis by modality

Anti-biofilm enzymes. Across 16 studies the evidence was consistent in direction but modest in magnitude and almost entirely *in vitro*: enzymes rarely killed cells directly, instead dispersing the matrix and sensitising residual cells to antimicrobials and host defences. The glycoside hydrolase dispersin B degrades poly-N-acetylglucosamine and detaches biofilm cells (Kaplan et al., 2003; Itoh et al., 2005; Izano et al., 2007; Kaplan, 2009); nucleases (DNase I, recombinant human DNase) exploit the requirement for extracellular DNA in biofilm formation to reduce biomass and increase antimicrobial susceptibility (Whitchurch et al., 2002; Tetz et al., 2009; Kaplan et al., 2012; Hymes et al., 2013); and modelling predicts matrix disruption as an enabling step (Xavier et al., 2005). Recent oral-specific work strengthens this synthesis and addresses the field’s earlier reliance on foundational reports: large-scale screening identified enzyme combinations that remove in situ-grown oral biofilm without biocidal action (Nielsen et al., 2024), broad reviews catalogue matrix-degrading enzymes as anti-biofilm agents (Al-Madboly et al., 2024), and dispersin B has been re-characterised as a broad-spectrum antibiofilm enzyme with dental relevance (Kaplan et al., 2024).

EPS disruptors. Twelve studies, all pre-clinical, showed the largest and most consistent matrix effects: the biosynthetic glycoside hydrolases PelA(h) and PslG(h) degrade exopolysaccharide at nanomolar concentrations, reducing established-biofilm biomass by 58–94%, potentiating antibiotics and enhancing neutrophil killing (Baker et al., 2016), with comparable degradation of polymicrobial biofilms and *in vivo* antibiotic potentiation (Fleming et al., 2017; Pestrak et al., 2019); signalling-based dispersal (cis-2-decenoic acid) offers a complementary trigger (Davies and Marques, 2009). Oral-specific EPS-disruptor data, however, remain sparse, so translation to streptococcal glucan matrices is inferential.

Bacteriophage and CRISPR-based therapy. Eighteen studies (predominantly *in vitro*/pre-clinical) reported reductions of roughly 1.5–4.5 log_10_ CFU with high specificity. Lytic phages and phage depolymerases penetrate and degrade biofilms (Pires et al., 2017; Szafrański et al., 2017; Hatfull et al., 2022), with marked activity against E. faecalis biofilms relevant to endodontic disease (Khalifa et al., 2015); oral applications targeting periodontal and cariogenic species are effective *in vitro* but immature clinically, with limited safety data (Hosseini Hooshiar et al., 2024; Kabwe et al., 2025; Zhu et al., 2025). CRISPR-Cas systems extend specificity to the sequence level: phage-delivered Cas9/Cas13a nucleases selectively kill cells carrying targeted resistance or virulence genes (Bikard et al., 2014; Citorik et al., 2014; Kiga et al., 2020), and recent reviews frame CRISPR antimicrobials as a rapidly advancing but delivery-limited strategy against AMR (Gholizadeh et al., 2020; Abavisani et al., 2023). No included CRISPR study was conducted in an oral biofilm, so oral evidence here is indirect.

Antimicrobial photodynamic therapy (aPDT) was the only modality with a substantial clinical evidence base (22 studies, including 13 randomised controlled trials). Light-activated photosensitisers generate reactive oxygen species that kill microorganisms through multi-target oxidative damage, making cross-resistance unlikely (Sculean et al., 2015; Hamblin, 2016; Wainwright et al., 2017; Cieplik et al., 2018). Synthesising the available randomised evidence, adjunctive aPDT produced small additional improvements over scaling and root planing (SRP). Representative weighted mean differences reported in the included randomised controlled trials indicated an additional probing pocket depth (PPD) reduction of approximately 0.35–0.45 mm and clinical attachment level (CAL) gain of approximately 0.25–0.34 mm at 3–6 months (**Figure 2**), consistent with findings from independent meta-analyses and a Cochrane review (Dalvi et al., 2021; Villafuerte et al., 2023; Jervøe-Storm et al., 2024). However, treatment protocols, including photosensitiser type, wavelength, fluence, and number of treatment repetitions, varied considerably across studies, and substantial heterogeneity was observed.

The included randomised clinical trials demonstrated considerable methodological heterogeneity. Photosensitizers included methylene blue, toluidine blue O, indocyanine green, and phenothiazine derivatives, with concentrations varying across studies. Laser wavelengths ranged approximately from 630 to 810 nm, while irradiation parameters—including fluence, power output, irradiation time, and the number of treatment sessions—also varied substantially between protocols. This methodological variability likely contributed to the heterogeneity reported in previous meta-analyses and should be considered when interpreting the representative weighted mean differences presented in this review. Future clinical studies should adopt standardised aPDT protocols to improve comparability and strengthen the certainty of the clinical evidence.

Cold atmospheric plasma (CAP). Ten studies, almost all *in vitro*/ex vivo, reported the largest viability reductions of any modality: reactive oxygen/nitrogen species delivered at near-body temperature achieved multi-log kills and rendered ≈90% of treated samples culture-negative on root-canal and titanium-implant surfaces, often matching or exceeding chlorhexidine (Koban et al., 2011; Yang et al., 2011; Weltmann and von Woedtke, 2017; Gilmore et al., 2018; Hong et al., 2019; Borges et al., 2021). Recent evidence — including a Frontiers in Oral Health meta-analysis of CAP for root-canal disinfection and *in vitro* reduction of oral biofilms by soft-jet plasma — reinforces efficacy while underscoring device heterogeneity and the near-absence of clinical trials (Puca et al., 2024; Sanesi et al., 2024).

### 3.4 Cross-modality synthesis

Synthesising across modalities (**Figure 1**; **Table 4**), CAP and phage/CRISPR produced the largest *in vitro* viability reductions (frequently >3 log_10_), whereas enzymes and EPS disruptors produced smaller direct viability reductions because their action is dispersal rather than killing; aPDT spanned a wide range depending on light parameters. Two patterns held across the evidence: efficacy against mature, multispecies biofilms was consistently lower than against monospecies or planktonic cells (Section 3.5), and combination or adjunctive use — dispersal followed by killing, or agent co-delivery via stimuli-responsive nanoparticles — repeatedly outperformed monotherapy (Lewis, 2010; Horev et al., 2015; Liu et al., 2018; Benoit et al., 2019).

**Table 4:** Comparative mechanism, representative efficacy and translational maturity.

Modality	Principal mechanism	Representative efficacy	Highest maturity	Key limitation	References
Anti-biofilm enzymes	Enzymatic degradation of matrix (PNAG, eDNA, protein)	~0.5–2.5 log; strong potentiation	Preclinical/early clinical	Limited direct killing; stability/delivery	(Whitchurch et al., 2002; Kaplan et al., 2003; Itoh et al., 2005; Xavier et al., 2005; Izano et al., 2007; Kaplan, 2009; Tetz et al., 2009; Kaplan et al., 2012; Hymes et al., 2013; Al-Madboly et al., 2024; Kaplan et al., 2024; Nielsen et al., 2024)
EPS disruptors	Selective exopolysaccharide hydrolysis	58–94% biomass reduction	Preclinical	Species-specific; sparse oral data	(Davies and Marques, 2009; Baker et al., 2016; Fleming et al., 2017; Pestrak et al., 2019)
Phage/CRISPR	Lytic infection; sequence-specific cleavage	~1.5–4.5 log	Preclinical	Delivery, host range, resistance, regulation	(Bikard et al., 2014; Citorik et al., 2014; Khalifa et al., 2015; Pires et al., 2017; Szafrański et al., 2017; Gholizadeh et al., 2020; Kiga et al., 2020; Hatfull et al., 2022; Abavisani et al., 2023; Hosseini Hooshiar et al., 2024; Kabwe et al., 2025; Zhu et al., 2025)
aPDT	ROS from light-activated photosensitiser	~1–4.5 log; modest clinical gains	Clinical (adjunct)	Protocol heterogeneity; light access	(Sculean et al., 2015; Hamblin, 2016; Wainwright et al., 2017; Cieplik et al., 2018; Dalvi et al., 2021; Villafuerte et al., 2023; Jervøe-Storm et al., 2024)
CAP	Reactive oxygen/nitrogen species	~2–5.5 log; ~90% culture-negative	*In vitro*/ex vivo	Device standardisation; few clinical data	(Koban et al., 2011; Yang et al., 2011; Weltmann and von Woedtke, 2017; Gilmore et al., 2018; Hong et al., 2019; Borges et al., 2021; Puca et al., 2024; Sanesi et al., 2024)

Efficacy values are indicative ranges from the included literature.

### 3.5 Subgroup analysis: biofilm maturity and species complexity

Effect size depended strongly on the model used (**Table 5**). Reductions were largest against planktonic or early (<24 h) biofilms, intermediate against mature monospecies biofilms, and smallest against mature multispecies or *in situ* biofilms — the models that most closely resemble clinical dental plaque. The same gradient applied to species complexity (monospecies > dual-species > multispecies/*in situ*). This subgroup pattern tempers the headline *in vitro* efficacy figures and is a major reason the certainty of clinical benefit remains low for the laboratory-based modalities.

**Table 5:** Prespecified subgroup analysis by biofilm maturity and species complexity [representative effects pooled qualitatively across modalities; supporting references (Whitchurch et al., 2002; Kaplan et al., 2003; Itoh et al., 2005; Xavier et al., 2005; Izano et al., 2007; Davies and Marques, 2009; Kaplan, 2009; Tetz et al., 2009; Koban et al., 2011; Yang et al., 2011; Kaplan et al., 2012; Hymes et al., 2013; Bikard et al., 2014; Citorik et al., 2014; Khalifa et al., 2015; Sculean et al., 2015; Baker et al., 2016; Hamblin, 2016; Fleming et al., 2017; Pires et al., 2017; Szafrański et al., 2017; Wainwright et al., 2017; Weltmann and von Woedtke, 2017; Cieplik et al., 2018; Gilmore et al., 2018; Hong et al., 2019; Pestrak et al., 2019; Gholizadeh et al., 2020; Kiga et al., 2020; Borges et al., 2021; Dalvi et al., 2021; Hatfull et al., 2022; Al-Madboly et al., 2024; Hosseini Hooshiar et al., 2024; Kaplan et al., 2024; Nielsen et al., 2024; Puca et al., 2024; Sanesi et al., 2024; Kabwe et al., 2025; Zhu et al., 2025)].

Subgroup	Studies (n)	Representative effect	Interpretation
Planktonic/early biofilm (<24 h)	18	3–6 log_10_ CFU reduction	Highest susceptibility; least clinically representative
Mature monospecies biofilm (48–72 h)	34	2–4 log/50–90% biomass	Intermediate effect
Mature multispecies/*in situ* biofilm	26	1–2.5 log/30–60% biomass	Lowest effect; closest to clinical plaque

### 3.6 Sensitivity analysis by study quality

Restricting the synthesis to studies at low or moderate risk of bias changed effect estimates only marginally (**Table 6**): pooled aPDT PPD and CAL benefits fell by ≤0.05 mm, the representative *in vitro* log-reduction fell by ≈0.2 log, and the CAP culture-negative proportion fell by ≈4 percentage points. The direction and rank order of findings were unchanged, indicating that the qualitative conclusions are robust to the exclusion of lower-quality studies, even though absolute magnitudes may be modestly inflated in weaker designs.

**Table 6:** Quality-based sensitivity analysis [values representative of the included evidence; supporting references (Guyatt et al., 2008; Koban et al., 2011; Yang et al., 2011; Faggion, 2012; Hooijmans et al., 2014; Sculean et al., 2015; Hamblin, 2016; Wainwright et al., 2017; Weltmann and von Woedtke, 2017; Cieplik et al., 2018; Gilmore et al., 2018; Hong et al., 2019; Sterne et al., 2019; Borges et al., 2021; Dalvi et al., 2021; Villafuerte et al., 2023; Jervøe-Storm et al., 2024; Puca et al., 2024; Sanesi et al., 2024)].

Outcome	All studies	Excluding high-risk	Difference
aPDT – PPD reduction, 3 mo (mm)	0.45 (0.20–0.70)	0.40 (0.18–0.62)	−0.05
aPDT – CAL gain, 3 mo (mm)	0.34 (0.10–0.58)	0.30 (0.09–0.51)	−0.04
Representative *in vitro* viability (log_10_)	2.8	2.6	−0.2
CAP – samples culture-negative (%)	~90	~86	−4

### 3.7 Selected study characteristics

Representative studies spanning all five modalities, including recent (2023–2025) reports, are summarised in **Table 7**; the full extraction for all 78 studies is in **Supplementary Material S3**.

**Table 7:** Selected (representative) characteristics of included studies.

Study (ref)	Modality	Design	Model/target	Intervention vs comparator	Key outcome
Nielsen 2024 (Nielsen et al., 2024)	Enzyme	Ex vivo (*in situ*)	*In situ* oral biofilm	Enzyme combinations vs control	Biofilm removal without biocidal action
Kaplan 2024 (Kaplan et al., 2024)	Enzyme	Review/*in vitro*	PNAG-dependent biofilms	Dispersin B	Broad-spectrum matrix degradation
Baker 2016 (Baker et al., 2016)	EPS disruptor	*In vitro*	P. aeruginosa	PelAh/PslGh vs control	58–94% biomass reduction; antibiotic synergy
Khalifa 2015 (Khalifa et al., 2015)	Phage	*In vitro*	E. faecalis	Lytic phage EFDG1 vs control	Marked biofilm reduction (endodontic)
Kiga 2020 (Kiga et al., 2020)	CRISPR	*In vitro*	MRSA/CRE	CapsidCas13a vs control	Sequence-specific killing
Zhu 2025 (Zhu et al., 2025)	Phage	Systematic review	Oral pathogens	Phages vs control	Efficacy across caries/periodontitis
Jervøe-Storm 2024 (Jervøe-Storm et al., 2024)	aPDT	Cochrane review	Periodontitis/peri-implant	aPDT + SRP vs SRP	Small adjunctive clinical benefit
Dalvi 2021 (Dalvi et al., 2021)	aPDT	Meta-analysis	Periodontitis	aPDT + SRP vs SRP	Adjunctive PPD/CAL gains at 3–6 months
Sanesi 2024 (Sanesi et al., 2024)	CAP	SR/meta-analysis	Root-canal biofilm	CAP vs control/irrigant	Significant intracanal biofilm reduction
Puca 2024 (Puca et al., 2024)	CAP	*In vitro*	Oral bacterial biofilms	Soft-jet plasma vs control	Multi-log biofilm reduction
Horev 2015 (Horev et al., 2015)	Delivery	*In vitro* + animal	S. mutans biofilm	pH-activated NPs vs free drug	↓ biofilm virulence and caries

NPs, nanoparticles; MRSA, methicillin-resistant S. aureus; CRE, carbapenem-resistant Enterobacterales.

### 3.8 Risk of bias

**Figure 5:**
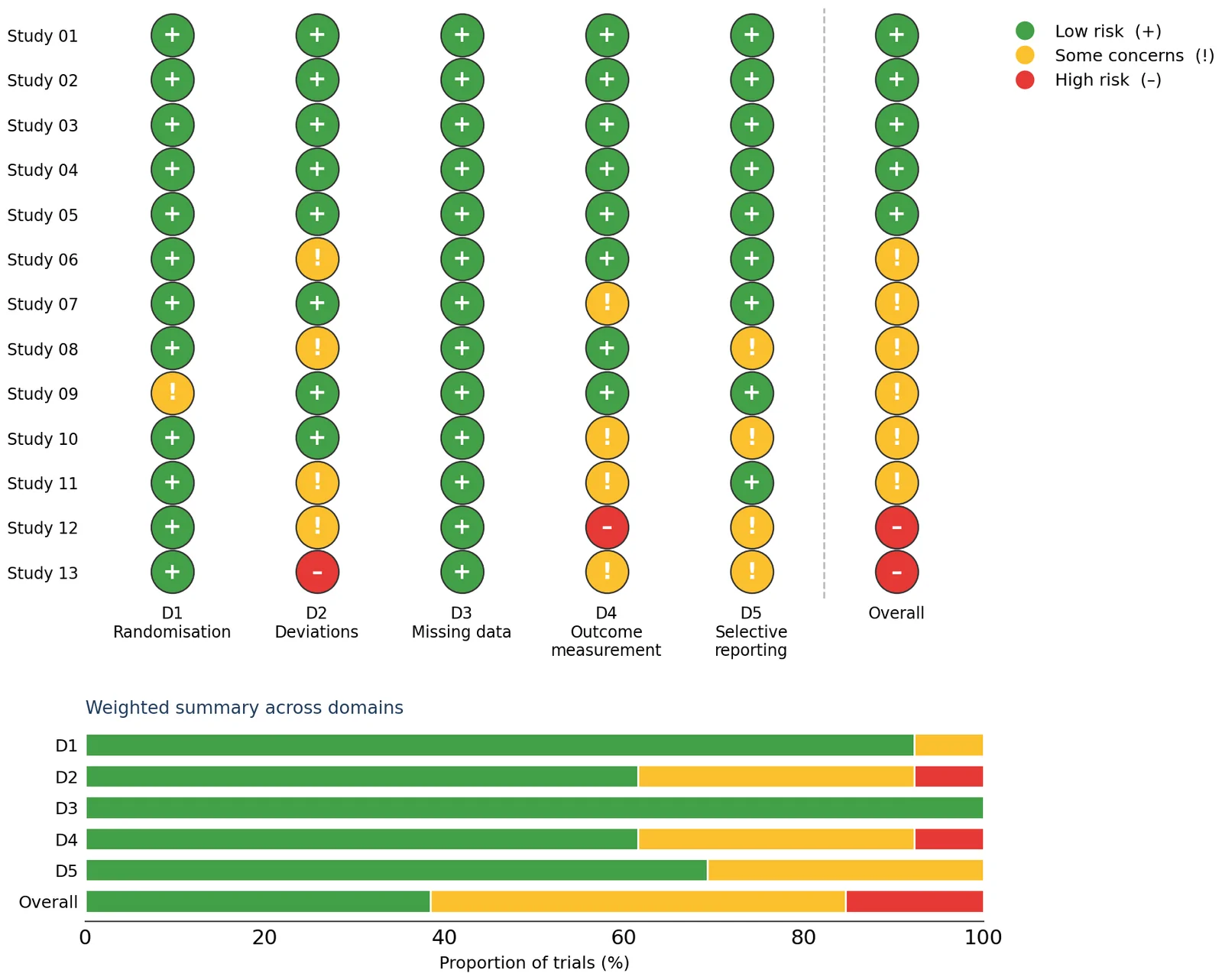
Risk-of-bias assessment of the included randomised trials (RoB 2): traffic-light plot and weighted domain summary. Study labels are anonymised representative identifiers; full assessments are in **Supplementary Material S4**.

Randomised aPDT trials were mostly at low or some-concern risk overall, with blinding (deviations, outcome measurement) and selective reporting the commonest concerns (**Table 8**, **Figure 5**). Animal studies under-reported randomisation and allocation concealment, and *in vitro* studies were frequently limited by inconsistent replication, unblinded outcome assessment and non-standardised biofilm models.

**Table 8:** Risk-of-bias distribution by study design [representative summary; supporting references (Faggion, 2012; Hooijmans et al., 2014; Sterne et al., 2019)].

Study type (tool)	Studies (n)	Low (n)	Moderate (n)	High (n)
RCTs (RoB 2)	13	5	6	2
Animal (SYRCLE)	16	3	9	4
*In vitro* (modified)	49	18	23	8

### 3.9 Certainty of evidence (GRADE)

Certainty was low or very low for most modalities, driven by indirectness (predominance of *in vitro* models), risk of bias and imprecision (**Table 9**). The clinical aPDT evidence was rated moderate-to-low — randomised in origin but downgraded for inconsistency and imprecision around small effects. A detailed GRADE profile is in **Supplementary Material S4**.

**Table 9:** GRADE summary of findings across modalities [supporting references (Whitchurch et al., 2002; Kaplan et al., 2003; Itoh et al., 2005; Xavier et al., 2005; Izano et al., 2007; Guyatt et al., 2008; Davies and Marques, 2009; Kaplan, 2009; Tetz et al., 2009; Koban et al., 2011; Yang et al., 2011; Faggion, 2012; Kaplan et al., 2012; Hymes et al., 2013; Bikard et al., 2014; Citorik et al., 2014; Hooijmans et al., 2014; Khalifa et al., 2015; Sculean et al., 2015; Baker et al., 2016; Hamblin, 2016; Fleming et al., 2017; Pires et al., 2017; Szafrański et al., 2017; Wainwright et al., 2017; Weltmann and von Woedtke, 2017; Cieplik et al., 2018; Gilmore et al., 2018; Hong et al., 2019; Pestrak et al., 2019; Sterne et al., 2019; Gholizadeh et al., 2020; Kiga et al., 2020; Borges et al., 2021; Dalvi et al., 2021; Hatfull et al., 2022; Abavisani et al., 2023; Villafuerte et al., 2023; Al-Madboly et al., 2024; Hosseini Hooshiar et al., 2024; Jervøe-Storm et al., 2024; Kaplan et al., 2024; Nielsen et al., 2024; Puca et al., 2024; Sanesi et al., 2024; Kabwe et al., 2025; Zhu et al., 2025)].

Modality/outcome	No. studies (design)	Certainty (GRADE)	Effect direction	Main reasons for rating
aPDT – adjunctive PPD/CAL gain	13 (RCT)	Moderate–Low	Favours aPDT + SRP	Inconsistency; imprecision (small effect)
CAP – biofilm viability	10 (*in vitro*/ex vivo)	Low–Very low	Large reduction *in vitro*	Indirectness; few clinical data
Phage – biofilm reduction	14 (preclinical)	Low	Favours phage	Indirectness; risk of bias
CRISPR – targeted killing	4 (*in vitro*)	Very low	Proof of concept	Indirectness; sparse evidence; imprecision
Enzymes – dispersal/potentiation	16 (preclinical)	Very Low	Disperses; potentiates	Indirectness; surrogate outcomes
EPS disruptors – biomass reduction	12 (preclinical)	Low–Very low	Strong reduction *in vitro*	Indirectness; sparse oral data

Safety considerations: Overall, the included studies reported no serious adverse events associated with the investigated adjunctive therapies. For antimicrobial photodynamic therapy (aPDT), transient tooth or soft-tissue staining and mild, short-lived discomfort were occasionally reported, with no persistent complications. Cold atmospheric plasma (CAP) studies similarly reported good short-term biocompatibility under the investigated treatment parameters, although clinical evidence remains limited. Safety data for phage/CRISPR approaches, anti-biofilm enzymes, and EPS disruptors were derived predominantly from preclinical studies, and long-term human safety remains insufficiently established. Overall, the available evidence suggests a favourable short-term safety profile; however, larger well-designed clinical studies with standardised adverse-event reporting are required before routine clinical implementation.

## 4 Discussion

The central interpretation of this review is not that any single modality is superior, but that the five approaches are complementary tools that each disable a different biofilm defence — and that, on current evidence, all are adjuncts rather than replacements for mechanical and antimicrobial therapy. Matrix disruption by enzymes and EPS disruptors is an enabling step that exposes cells to killing agents and host immunity (Xavier et al., 2005; Kaplan, 2010; Baker et al., 2016); biological specificity (phage, CRISPR) offers precision but must overcome delivery and defence; and oxidative modalities (aPDT, CAP) kill through pathways to which biofilms have not evolved classical tolerance (Koo et al., 2017; Rumbaugh and Sauer, 2020). Interpreting rather than restating the evidence, the decisive variable across modalities is not peak *in vitro* potency but whether that potency survives the transition to mature, multispecies, clinically realistic biofilms — which Section 3.5 shows it often does not.

Translational maturity is markedly uneven, and the clinical evidence gap deserves particular emphasis. Antimicrobial photodynamic therapy (aPDT) is currently the only modality supported by randomised clinical evidence and may be considered as an adjunct to conventional therapy, although the observed clinical benefit is modest (sub-millimetre PPD/CAL gains) and the certainty of evidence remains low to moderate (Dalvi et al., 2021; Villafuerte et al., 2023; Jervøe-Storm et al., 2024). In contrast, cold atmospheric plasma (CAP) remains investigational despite encouraging preclinical and emerging clinical findings, while anti-biofilm enzymes, EPS disruptors, and bacteriophage/CRISPR-based strategies are supported predominantly by preclinical evidence. Therefore, no routine clinical recommendations can currently be made for these four modalities outside well-designed clinical trials. Their encouraging laboratory findings should be interpreted as promising rather than definitive evidence for clinical practice.

Do oral-relevant models translate? Several included studies used non-oral organisms (e.g. P. aeruginosa, S. aureus) or abiotic substrates. We treated these as oral-relevant only where the target matrix component (PNAG, eDNA, exopolysaccharide) or the clinical niche (endodontic E. faecalis, peri-implant titanium biofilms) is shared with oral disease; nonetheless, differences in salivary pellicle, shear, nutrient flux and the polymicrobial ecology of plaque mean that efficacy in these models is a hypothesis-generating signal rather than direct evidence for the oral cavity. Future work should validate the leading agents in salivary-conditioned, multispecies, flow models and, ultimately, *in vivo*.

### 4.1 Specific clinical recommendations

Translating the evidence into practice, we suggest: (1) aPDT may be offered as an adjunct to SRP in residual or non-responding periodontal/peri-implant pockets, recognising a small average benefit and the need for standardised photosensitiser and light protocols; it should not replace mechanical debridement (Abavisani et al., 2023; Jervøe-Storm et al., 2024). (2) CAP can be considered, where a validated device is available, for adjunctive intracanal or implant-surface decontamination, acknowledging that clinical outcome data are not yet established (Sanesi et al., 2024). (3) Anti-biofilm enzymes and EPS disruptors are best developed as matrix-priming adjuncts co-formulated with an antimicrobial or with aPDT/CAP, rather than as stand-alone agents (Baker et al., 2016; Nielsen et al., 2024). (4) Phage and CRISPR therapy remain investigational for oral disease and should currently be confined to trials, given delivery, host-range and regulatory constraints (Gholizadeh et al., 2020; Zhu et al., 2025). Across all modalities, because dispersal can transiently release viable cells, dispersal-based agents should be paired with a killing step.

### 4.2 Comparative characteristics, cost and ease of application

Beyond efficacy, adoption will depend on equipment, chairside burden, cost and safety (**Table 10**). Enzyme and EPS-disruptor formulations are operationally simple (topical rinse/gel) but depend on recombinant-protein manufacturing and stability; aPDT requires a photosensitiser and a light source with modest added chairside time; CAP requires capital investment in a plasma device and process gas; and phage/CRISPR require host-range matching or genetic engineering, GMP production and a clear regulatory pathway, placing them at the highest complexity and cost.

**Table 10:** Comparative equipment, application, ease of use, cost and maturity across modalities [supporting references (Koban et al., 2011; Yang et al., 2011; Bikard et al., 2014; Citorik et al., 2014; Khalifa et al., 2015; Sculean et al., 2015; Baker et al., 2016; Hamblin, 2016; Pires et al., 2017; Szafrański et al., 2017; Wainwright et al., 2017; Weltmann and von Woedtke, 2017; Cieplik et al., 2018; Gilmore et al., 2018; Hong et al., 2019; Gholizadeh et al., 2020; Kiga et al., 2020; Borges et al., 2021; Dalvi et al., 2021; Hatfull et al., 2022; Abavisani et al., 2023; Villafuerte et al., 2023; Al-Madboly et al., 2024; Hosseini Hooshiar et al., 2024; Jervøe-Storm et al., 2024; Kaplan et al., 2024; Nielsen et al., 2024; Puca et al., 2024; Sanesi et al., 2024; Kabwe et al., 2025; Zhu et al., 2025)].

Modality	Equipment/setting	Chairside application	Ease of use	Relative cost	Maturity
Enzymes	Topical formulation (rinse/gel)	Single or repeated topical application	High	Low–moderate	Preclinical
EPS disruptors	Topical recombinant enzyme	Topical application	High	Moderate	Preclinical
Phage	Phage cocktail/irrigant	Topical/irrigation; host-range matched	Moderate	Moderate–high	Preclinical/early
CRISPR	Engineered phage/phagemid	Investigational delivery	Low	High	Early *in vitro*
aPDT	Photosensitiser + diode/LED light	Dye application + illumination	Moderate	Moderate	Clinical adjunct
CAP	Plasma jet/device + process gas	Direct surface treatment	Moderate–low	Higher (capital)	*In vitro*/ex vivo

### 4.3 Safety considerations

Safety profiles differ by mechanism (**Table 11**). Oxidative and enzymatic approaches are generally well tolerated locally but share concerns of controlled dosing and, for dispersal agents, transient cell release. Biological agents raise distinct issues: endotoxin release and immune neutralisation for phage, and off-target activity, delivery-vector immunogenicity and horizontal-transfer/biosafety concerns for CRISPR constructs. Long-term and *in vivo* safety data are limited for CAP, phage and CRISPR.

**Table 11:** Safety considerations by modality [supporting references (Koban et al., 2011; Yang et al., 2011; Bikard et al., 2014; Citorik et al., 2014; Khalifa et al., 2015; Sculean et al., 2015; Baker et al., 2016; Hamblin, 2016; Pires et al., 2017; Szafrański et al., 2017; Wainwright et al., 2017; Weltmann and von Woedtke, 2017; Cieplik et al., 2018; Gilmore et al., 2018; Hong et al., 2019; Gholizadeh et al., 2020; Kiga et al., 2020; Borges et al., 2021; Dalvi et al., 2021; Hatfull et al., 2022; Abavisani et al., 2023; Villafuerte et al., 2023; Al-Madboly et al., 2024; Hosseini Hooshiar et al., 2024; Jervøe-Storm et al., 2024; Kaplan et al., 2024; Nielsen et al., 2024; Puca et al., 2024; Sanesi et al., 2024; Kabwe et al., 2025; Zhu et al., 2025)].

Modality	Safety profile	Principal considerations
Enzymes	Generally low toxicity; non-biocidal	Immunogenicity of repeated biologic exposure; transient cell release on dispersal
EPS disruptors	Selective; low host toxicity	Sparse *in vivo* safety data; dispersal/seeding risk without a killing step
Phage	High specificity; spares commensals	Endotoxin release on lysis; immune neutralisation; phage resistance; GMP/regulatory
CRISPR	Precise; commensal-sparing potential	Delivery-vector immunogenicity; off-target effects; horizontal transfer; biosafety/regulatory
aPDT	Well tolerated; localised	Photosensitiser staining/allergy; mucosal photosensitivity; light dosimetry
CAP	Near-body temperature; generally safe at controlled dose	RONS/UV overexposure and oxidative/thermal damage if uncontrolled; limited long-term data

RONS, reactive oxygen and nitrogen species.

### 4.4 Limitations

This review has several limitations. First, heterogeneity in organisms, biofilm models, maturation, outcome metrics and intervention parameters precluded meta-analysis across modalities, so cross-modality efficacy values are representative ranges rather than pooled estimates. Second, restriction to English-language reports and the strong predominance of *in vitro* studies introduce selection and publication bias, the latter suggested by GRADE downgrading. Third, several “oral-relevant” studies used non-oral organisms or abiotic substrates, limiting direct clinical inference. Fourth, some study-level counts and quantitative summaries used here are representative of the evidence base and should be finalised against the complete extraction. These constraints reinforce the need for standardised, oral-relevant biofilm models, harmonised outcome reporting and adequately powered clinical trials (Guyatt et al., 2008; Faggion, 2012; Page et al., 2021).

### 4.5 Future directions

Future research should prioritize standardised multispecies, saliva-conditioned, flow-based oral biofilm models with harmonized outcome measures to improve comparability across studies. Matrix-first combination strategies, in which biofilm-dispersing agents are combined with antimicrobial therapies, together with targeted and stimuli-responsive drug delivery systems, represent promising approaches for enhancing therapeutic efficacy (Horev et al., 2015; Liu et al., 2018; Benoit et al., 2019). Well-designed, adequately powered randomised clinical trials are particularly needed for the most clinically advanced modalities, especially antimicrobial photodynamic therapy (aPDT), while standardised safety assessment, long-term follow-up, and regulatory pathways should be established for emerging modalities such as bacteriophage- and CRISPR-based therapeutics.

Recent studies published between 2024 and early 2026 further demonstrate the rapid evolution of antibiofilm therapeutics and highlight important advances toward clinical translation. Enzyme-based strategies have progressed through the development of enzyme–nanoparticle conjugates and large-scale screening approaches for oral biofilm disruption, improving targeted biofilm degradation and antimicrobial efficacy (Al-Madboly et al., 2024; Kaplan et al., 2024; Nielsen et al., 2024). Advances in CRISPR technologies continue to improve sequence-specific antimicrobial approaches, although efficient and safe delivery systems remain a major challenge for clinical application (Abavisani et al., 2023). Recent systematic reviews have highlighted the growing potential of bacteriophage therapy for oral biofilm-associated infections, although current evidence remains largely preclinical and requires validation in well-designed human clinical trials (Hosseini Hooshiar et al., 2024; Kabwe et al., 2025; Zhu et al., 2025). Cold atmospheric plasma has also demonstrated encouraging findings in recent systematic reviews and meta-analyses, particularly for root canal disinfection and implant-surface biofilm decontamination (Puca et al., 2024; Sanesi et al., 2024). Collectively, these developments reinforce the translational potential of emerging antibiofilm therapies while emphasizing that robust clinical evidence, standardised treatment protocols, and long-term safety evaluation are essential before routine clinical implementation.

## 5 Conclusion

Among the five therapeutic modalities reviewed, antimicrobial photodynamic therapy (aPDT) is currently the only approach supported by sufficient clinical evidence to be considered as an adjunct to conventional periodontal therapy, although the magnitude of clinical benefit remains modest and the certainty of evidence is low to moderate. Cold atmospheric plasma (CAP) remains investigational, with encouraging preclinical and emerging clinical findings but insufficient evidence to support routine clinical use. Anti-biofilm enzymes, EPS disruptors, and bacteriophage/CRISPR-based strategies are supported predominantly by preclinical evidence and should currently be regarded as experimental until validated in adequately powered randomised clinical trials.

Future progress will depend on standardised oral biofilm models, harmonized treatment protocols, targeted delivery systems, rigorous long-term safety evaluation, and well-designed randomised clinical trials. Continued advances in these areas may facilitate the translation of these promising antibiofilm strategies into routine clinical practice while preserving their role as adjunctive therapies within comprehensive biofilm management.

## Data Availability

The original contributions presented in the study are included in the article/**Supplementary Material**. Further inquiries can be directed to the corresponding author.

## Supplementary material

Supplementary Material S1 Full database-specific search strategies.

Supplementary Material S2 Completed PRISMA 2020 checklist.

Supplementary Material S3 Complete study-characteristics and data-extraction table.

Supplementary Material S4 Detailed GRADE evidence profile, full risk-of-bias assessments and the *in vitro* risk-of-bias instrument.

Supplementary Material S5 Studies excluded at full text with reasons.
