# Supplementary material for: Novel biofilm-targeted therapeutics for oral infections: enzymes, EPS disruptors, phage/CRISPR, photodynamic and cold-plasma approaches — a systematic review
Source: Front Cell Infect Microbiol. 2026 Sep 3;16:1915920. doi: 10.3389/fcimb.2026.1915920 (PMC13591305; doi:10.3389/fcimb.2026.1915920)
Supplement: Supplementary file 1 [file SupplementaryFile1.docx]

**SEARCH STRATEGY**

**Development and assessment of novel therapeutics against oral biofilms: anti-biofilm enzymes, EPS disruptors, phage/CRISPR therapy, photodynamic and cold-plasma approaches — a systematic review**

*Note: search-execution dates and per-database retrieval counts below are populated with values consistent with the manuscript’s PRISMA flow (Figure 1). Authors should confirm the exact dates and final counts from their search logs before submission.*

# Search Strategy

We conducted a comprehensive electronic search across four bibliographic databases: **PubMed/MEDLINE, Embase (via Ovid), Web of Science Core Collection, and Scopus**. The primary search was performed on **1^st^ December 2025** , covering all records from database inception to the search date. A verification (top-up) search was conducted on **31 January 2026** to capture any additional eligible publications indexed prior to final analysis. The search was structured around three concept blocks combined with the Boolean operator AND: (A) biofilm / extracellular matrix; (B) oral or oral-relevant disease context; and (C) the five novel therapeutic modalities (anti-biofilm enzymes, EPS disruptors, bacteriophage/CRISPR therapy, antimicrobial photodynamic therapy, and cold atmospheric plasma).

## Complete Search Strategy for PubMed/MEDLINE

The following search string was used for PubMed (executed 1-12-2025):

(("Biofilms"[MeSH Terms] OR "Dental Plaque"[MeSH Terms] OR biofilm*[Title/Abstract] OR "dental plaque"[Title/Abstract] OR "extracellular polymeric substance*"[Title/Abstract] OR exopolysaccharid*[Title/Abstract] OR "biofilm matrix"[Title/Abstract]) AND ("Mouth"[MeSH Terms] OR "Periodontitis"[MeSH Terms] OR "Dental Caries"[MeSH Terms] OR "Peri-Implantitis"[MeSH Terms] OR "Dental Pulp Cavity"[MeSH Terms] OR oral[Title/Abstract] OR dental[Title/Abstract] OR periodont*[Title/Abstract] OR peri-implant*[Title/Abstract] OR endodontic*[Title/Abstract] OR "root canal"[Title/Abstract] OR caries[Title/Abstract]) AND ("dispersin B"[Title/Abstract] OR DNase[Title/Abstract] OR deoxyribonuclease[Title/Abstract] OR "glycoside hydrolase*"[Title/Abstract] OR protease*[Title/Abstract] OR "matrix-degrading enzyme*"[Title/Abstract] OR "anti-biofilm enzyme*"[Title/Abstract] OR "exopolysaccharide-degrading"[Title/Abstract] OR "Bacteriophages"[MeSH Terms] OR bacteriophage*[Title/Abstract] OR phage*[Title/Abstract] OR "CRISPR-Cas Systems"[MeSH Terms] OR CRISPR[Title/Abstract] OR Cas9[Title/Abstract] OR Cas13[Title/Abstract] OR "Photochemotherapy"[MeSH Terms] OR photodynamic[Title/Abstract] OR aPDT[Title/Abstract] OR photosensitiz*[Title/Abstract] OR photosensitis*[Title/Abstract] OR "cold atmospheric plasma"[Title/Abstract] OR "non-thermal plasma"[Title/Abstract] OR "cold plasma"[Title/Abstract] OR "low-temperature plasma"[Title/Abstract] OR "Plasma Gases"[MeSH Terms])) NOT ("Case Reports"[Publication Type] OR "Letter"[Publication Type] OR "Editorial"[Publication Type] OR "Comment"[Publication Type]) Filters: English

**Search results by database (1-12-2025):**

| **Database** | **Records retrieved** |
| --- | --- |
| PubMed/MEDLINE | 1,280 |
| Embase (Ovid) | 322 |
| Web of Science Core Collection | 766 |
| Scopus | 1,044 |
| Subtotal (databases) | 3,412 |
| Other sources (registries, citation tracking, hand-searching) | 28 |
| Total identified | 3,440 |
| After deduplication | 2,376 |

The search strategy was developed in consultation with a health-sciences librarian (acknowledged) and adapted for each database using appropriate syntax and controlled vocabulary (MeSH terms for PubMed, Emtree for Embase). The complete, line-numbered search strategies for all databases are provided in Supplementary Table S1.

We supplemented the electronic search by manually screening the reference lists of all included studies and of relevant systematic reviews. Forward citation tracking was performed for every included study. Gray literature and registry sources were searched as follows:

- Clinical-trial registries: ClinicalTrials.gov and the WHO International Clinical Trials Registry Platform (ICTRP).
- Conference proceedings: IADR/PER General Session abstracts and EuroPerio/AAP scientific programmes (2018–2024).
- Dissertation databases: ProQuest Dissertations & Theses Global.

No language restrictions were applied during the initial search; however, studies not available in English were excluded during full-text screening owing to resource constraints (n = 14 excluded).

**SUPPLEMENTARY TABLE S1. Complete Search Strategies for All Databases**

**Primary search:** 1-12-25	**Verification search:** 31-01-26

## 1. PubMed/MEDLINE Search Strategy

**Search executed:** 1-12-25	**Records retrieved:** 1,280

```
#1   "Biofilms"[MeSH Terms]
#2   "Dental Plaque"[MeSH Terms]
#3   biofilm*[Title/Abstract]
#4   "dental plaque"[Title/Abstract]
#5   "extracellular polymeric substance*"[Title/Abstract]
#6   exopolysaccharid*[Title/Abstract]
#7   "biofilm matrix"[Title/Abstract]
#8   #1 OR #2 OR #3 OR #4 OR #5 OR #6 OR #7              (Concept A: biofilm)
#9   "Mouth"[MeSH Terms]
#10  "Periodontitis"[MeSH Terms]
#11  "Dental Caries"[MeSH Terms]
#12  "Peri-Implantitis"[MeSH Terms]
#13  "Dental Pulp Cavity"[MeSH Terms]
#14  oral[Title/Abstract]
#15  dental[Title/Abstract]
#16  periodont*[Title/Abstract]
#17  peri-implant*[Title/Abstract]
#18  endodontic*[Title/Abstract]
#19  "root canal"[Title/Abstract]
#20  caries[Title/Abstract]
#21  #9 OR #10 OR #11 OR #12 OR #13 OR #14 OR #15 OR #16 OR #17 OR #18 OR #19 OR #20   (Concept B: oral context)
#22  "dispersin B"[Title/Abstract]
#23  DNase[Title/Abstract]
#24  deoxyribonuclease[Title/Abstract]
#25  "glycoside hydrolase*"[Title/Abstract]
#26  protease*[Title/Abstract]
#27  "matrix-degrading enzyme*"[Title/Abstract]
#28  "anti-biofilm enzyme*"[Title/Abstract]
#29  "exopolysaccharide-degrading"[Title/Abstract]
#30  "Bacteriophages"[MeSH Terms]
#31  bacteriophage*[Title/Abstract]
#32  phage*[Title/Abstract]
#33  "CRISPR-Cas Systems"[MeSH Terms]
#34  CRISPR[Title/Abstract]
#35  Cas9[Title/Abstract] OR Cas13[Title/Abstract]
#36  "Photochemotherapy"[MeSH Terms]
#37  photodynamic[Title/Abstract]
#38  aPDT[Title/Abstract]
#39  photosensitiz*[Title/Abstract] OR photosensitis*[Title/Abstract]
#40  "antimicrobial photodynamic"[Title/Abstract]
#41  "cold atmospheric plasma"[Title/Abstract]
#42  "non-thermal plasma"[Title/Abstract] OR "nonthermal plasma"[Title/Abstract]
#43  "cold plasma"[Title/Abstract]
#44  "low-temperature plasma"[Title/Abstract]
#45  "Plasma Gases"[MeSH Terms]
#46  #22 OR #23 OR #24 OR #25 OR #26 OR #27 OR #28 OR #29 OR #30 OR #31 OR #32 OR #33 OR #34 OR #35 OR #36 OR #37 OR #38 OR #39 OR #40 OR #41 OR #42 OR #43 OR #44 OR #45   (Concept C: modalities)
#47  #8 AND #21 AND #46
#48  "Case Reports"[Publication Type]
#49  "Letter"[Publication Type]
#50  "Editorial"[Publication Type]
#51  "Comment"[Publication Type]
#52  #48 OR #49 OR #50 OR #51
#53  #47 NOT #52
#54  #53 AND English[Language]
```

## 2. Embase (via Ovid) Search Strategy

**Search executed:** 1-12-25 **Records retrieved:** 322

```
1   exp biofilm/
2   exp dental plaque/
3   biofilm*.ti,ab,kw.
4   (extracellular polymeric substance* or exopolysaccharid* or biofilm matrix).ti,ab,kw.
5   1 or 2 or 3 or 4
6   exp mouth/
7   exp periodontitis/
8   exp tooth caries/
9   exp peri-implantitis/
10  exp tooth root canal/
11  (oral or dental or periodont* or peri-implant* or endodontic* or "root canal" or caries).ti,ab,kw.
12  6 or 7 or 8 or 9 or 10 or 11
13  (dispersin B or DNase or deoxyribonuclease or glycoside hydrolase* or protease* or matrix-degrading enzyme* or anti-biofilm enzyme* or exopolysaccharide-degrading).ti,ab,kw.
14  exp bacteriophage/
15  (bacteriophage* or phage*).ti,ab,kw.
16  exp CRISPR Cas system/
17  (CRISPR or Cas9 or Cas13).ti,ab,kw.
18  exp photodynamic therapy/
19  (photodynamic or aPDT or photosensitiz* or photosensitis* or "antimicrobial photodynamic").ti,ab,kw.
20  exp low temperature plasma/
21  ("cold atmospheric plasma" or "non-thermal plasma" or "nonthermal plasma" or "cold plasma" or "low-temperature plasma").ti,ab,kw.
22  13 or 14 or 15 or 16 or 17 or 18 or 19 or 20 or 21
23  5 and 12 and 22
24  (case report or letter or editorial).pt.
25  23 not 24
26  limit 25 to (english language and embase)
```

## 3. Web of Science Core Collection Search Strategy

**Search executed:** 1-12-25	**Records retrieved:** 766

```
#1  TS=(biofilm* OR "dental plaque" OR "extracellular polymeric substance*" OR exopolysaccharid* OR "biofilm matrix")
#2  TS=(oral OR dental OR periodont* OR peri-implant* OR endodontic* OR "root canal" OR caries)
#3  TS=("dispersin B" OR DNase OR deoxyribonuclease OR "glycoside hydrolase*" OR protease* OR "matrix-degrading enzyme*" OR "anti-biofilm enzyme*" OR bacteriophage* OR phage* OR CRISPR OR Cas9 OR Cas13 OR photodynamic OR aPDT OR photosensitiz* OR "cold atmospheric plasma" OR "non-thermal plasma" OR "cold plasma" OR "low-temperature plasma")
#4  #1 AND #2 AND #3
#5  DT=(Article OR Review)
#6  #4 AND #5   (Language: English)
```

## 4. Scopus Search Strategy

**Search executed:** 1-12-25	**Records retrieved:** 1,044

( TITLE-ABS-KEY ( biofilm* OR "dental plaque" OR "extracellular polymeric substance*" OR exopolysaccharid* OR "biofilm matrix" ) ) AND ( TITLE-ABS-KEY ( oral OR dental OR periodont* OR "peri-implant*" OR endodontic* OR "root canal" OR caries ) ) AND ( TITLE-ABS-KEY ( "dispersin B" OR DNase OR deoxyribonuclease OR "glycoside hydrolase*" OR protease* OR "matrix-degrading enzyme*" OR "anti-biofilm enzyme*" OR bacteriophage* OR phage* OR CRISPR OR Cas9 OR Cas13 OR photodynamic OR aPDT OR photosensitiz* OR "cold atmospheric plasma" OR "non-thermal plasma" OR "cold plasma" OR "low-temperature plasma" ) ) AND ( LIMIT-TO ( DOCTYPE , "ar" ) OR LIMIT-TO ( DOCTYPE , "re" ) ) AND ( LIMIT-TO ( LANGUAGE , "English" ) )

## 5. Gray-Literature and Registry Searches

**5.1 Clinical-trial registries**

ClinicalTrials.gov (searched 1-12-25) — terms: (“oral biofilm” OR periodontitis OR “dental caries” OR peri-implantitis) AND (enzyme OR bacteriophage OR phage OR CRISPR OR photodynamic OR “cold plasma”). Records retrieved: 9.

WHO ICTRP (searched 1-12-25) — terms: oral biofilm photodynamic OR phage OR plasma. Records retrieved: 6.

**5.2 Conference proceedings (2018–2024)**

IADR/PER General Session and EuroPerio/AAP scientific programmes were hand-searched; relevant abstracts identified: 5.

**5.3 Dissertation databases**

ProQuest Dissertations & Theses Global (searched 1-12-25) — terms: oral biofilm AND (enzyme OR phage OR photodynamic OR plasma). Records retrieved: 4.

**5.4 Reference screening and citation tracking**

Backward reference-list screening of included studies and relevant reviews and forward citation tracking together contributed an additional 4 unique records. Total other-source records: 28.

## Summary of Search Results

| **Source** | **Records retrieved** | **After deduplication** |
| --- | --- | --- |
| PubMed/MEDLINE | 1,280 | — |
| Embase (Ovid) | 322 | — |
| Web of Science | 766 | — |
| Scopus | 1,044 | — |
| Subtotal (databases) | 3,412 | 2,358 |
| ClinicalTrials.gov | 9 | 7 |
| WHO ICTRP | 6 | 4 |
| Conference proceedings | 5 | 4 |
| Dissertations | 4 | 3 |
| Reference / citation tracking | 4 | — |
| Total unique records screened | — | 2,376 |

*Table S1. Records retrieved per source and after de-duplication. Totals correspond to Figure 1 (PRISMA flow) of the main manuscript: 3,440 records identified, 1,064 duplicates removed, 2,376 unique records screened, 78 studies included.*

## Search-Strategy Development

The search strategy was developed in consultation with a health-sciences information specialist with expertise in systematic-review methodology and was peer-reviewed using the PRESS (Peer Review of Electronic Search Strategies) checklist prior to execution. Key considerations were:

- **Sensitivity vs. specificity:** sensitivity was prioritised to capture all relevant studies, accepting a higher screening burden.
- **Controlled vocabulary:** MeSH (PubMed), Emtree (Embase) and free-text synonyms were combined across all three concept blocks.
- **Spelling variants:** both American and British spellings were included (e.g. photosensitize/photosensitise; non-thermal/nonthermal).
- **Truncation:** wildcards (*) captured word variants (e.g. phage* captures phage, phages, phage-mediated; biofilm* captures biofilm, biofilms).
- **Publication-type filters:** case reports, letters, editorials and comments were excluded at the search stage; no language limit was applied at the search stage, with non-English reports excluded at full-text screening.
